# Associations between community-based aged care services and subjective well-being among older adults in China: the mediating roles of self-rated and mental health

**DOI:** 10.1371/journal.pone.0341877

**Published:** 2026-04-06

**Authors:** Ya Wang, Xiaojing Bai, Haitang Zhao

**Affiliations:** College of Marxism, Chengdu University of Technology, Chengdu, People’s Republic of China; UofM: The University of Memphis, UNITED STATES OF AMERICA

## Abstract

**Background:**

Against the backdrop of accelerating population aging worldwide, promoting well-being among older populations has emerged as a critical objective for achieving healthy aging. Community-based aged care services (CACS) represent a key strategy to complement informal kinship-based support and strengthen the comprehensive old-age welfare system.

**Methods:**

This study utilized cross-sectional data from the nationally representative 2018 Chinese Longitudinal Healthy Longevity Survey (CLHLS) to explore the relationship between the availability of CACS and the subjective well-being of older adults. We employed an ordinary least squares (OLS) regression model to examine this association, followed by a Bootstrap mediation analysis to test the potential mediating roles of self-rated health and mental health.

**Results:**

The empirical findings indicate that a greater availability of CACS is significantly associated with higher levels of subjective well-being among older adults, and this association varied by gender, age, region, and the specific type of CACS service utilized. Furthermore, self-rated health and mental health were identified as significant mediators in the relationship between CACS availability and subjective well-being.

**Conclusion:**

The study concludes that the availability of CACS is positively linked to the subjective well-being of older adults, partly through better self-rated health and mental health. These findings suggest that with the support of CACS, older adults may be better equipped to adapt to the aging process and maintain a positive outlook on life.

## 1 Introduction

With the acceleration of global population aging, the issue of old age has become a social issue of common concern to all countries and has been incorporated into the policy agenda [1]. For the increasing number of older adults, subjective well-being, as a key indicator of their quality of life, is not only related to the physical and mental health of individuals, but also to the overall harmony and stability of society. China has entered a moderately aging society, with the older adult population aged 60 and above exceeding 264 million in 2023, accounting for more than 20% of the total population. The population aged 65 and above exceeding 191 million, accounting for about 13.5% of the total population [2,3]. Under the traditional Chinese model of “raising children for old age”, family support plays an absolutely dominant role in enhancing the well-being of older adults. However, with the mobility of the labor force and the miniaturization of family size, the traditional family model of old-age care has been impacted, and the function of family in old age has continued to weaken, so that older adults are easily caught in the predicament of poor life care and lack of spiritual solace. In order to fill the gaps and deficiencies in family care services and cope with the expanding risks of old-age care, the vigorous development of community-based aged care services (CACS) has become the policy direction for China’s response to population ageing [4].

CACS, defined as daily living assistance provided by governmental and societal entities to older adults, serve as a critical mechanism for facilitating aging in place [5]. Existing literature has primarily investigated CACS through two lenses: demand-side determinants and outcome evaluation. First, based on the research data, it has been found that older adults generally have a strong demand for community-based aged care services, especially in the areas of life care and spiritual comfort [6,7]. In terms of influencing factors, individual factors such as education level and health condition, internal family factors such as number of kids and lifestyle, and external social environment factors such as the level of socio-economic, development of the region and the social security system for older adults are the important factors affecting the demand for CACS [8,9]. Second, previous studies have assessed the functionalities of CACS from different perspectives of life satisfaction, quality of life, and physical and psychological health of older people. The empirical results have shown that CACS have a significant positive influence on safeguarding the wellness of seniors and improving their life satisfaction [10–14].

Despite existing advancements, critical research gaps remain to be addressed. Prior research has often focused on service availability; however, there is a critical need to investigate aspects of service quality and utilization. As a pivotal component of the care support system for older adults, CACS have predominantly been evaluated through metrics such as life satisfaction and quality of life in prior studies, while a comprehensive assessment of subjective well-being remains underexplored. More importantly, the mechanisms underlying the relationship between CACS and well-being lack systematic empirical validation.

To explore these questions, this study establishes subjective well-being as a core variable and investigates the relationship between CACS availability and well-being. Grounded in social support theory, it further elucidates the potential underlying mechanisms through two health-related pathways: self-rated health and mental health, which represent core components of overall psychological well-being.

Using the Chinese Longitudinal Healthy Longevity Survey (CLHLS) 2018 data, this study examines the association between CACS availability and older adults’ subjective well-being. Its innovations are threefold: First, prior research utilizing the CLHLS has predominantly focused on objective health outcomes or generic life satisfaction, this study provides a novel empirical investigation into the link between CACS and a multidimensional construct of subjective well-being. Second, it moves beyond establishing a direct link to systematically test and validate the parallel mediating roles of self-rated health and mental health. Third, by identifying which specific service types show the strongest associations and for which subpopulations, our findings offer targeted, actionable pathways for optimizing community care systems, thereby providing nuanced, evidence-based solutions to aging-population challenges.

The components of this study are as follows. Section 2 formulates the research hypotheses and develops the theoretical framework. Section 3 specifies the data sources, operational variables and analytical methods. Section 4 presents the main empirical results, while section 5 critically interprets the results, discusses the policy implications, makes recommendations and acknowledges the limitations. Finally, section 6 summarizes the main contributions of the study.

## 2 Research hypothesis

Extensive empirical evidence has shown that social support significantly contributes the subjective well-being of seniors [15–17]. Defined as the assistance and resources individuals obtain from their social networks [18], social support operates through a tripartite structure comprising inner, middle, and outer layers. The innermost layer represents intimate relational support (e.g., family and close friends), the intermediate layer encompasses social network support (e.g., peers and organizations), while the outermost layer corresponds to community-level support [19]. Within this framework, China has strategically prioritized community support as a critical pillar of its social support system. Since initiating the National Aging Policy (1994), China has positioned communities as hubs for integrating public resources, catalyzing nationwide development of CACS [20,21]. As an institutionalized form of community support in geriatric healthcare, CACS aligns with social support theory by addressing older adults’ care needs and amplifying their well-being. Therefore, we propose the following hypothesis:

H1: CACS are positively associated with the subjective well-being of older adults.

CACS serve as a critical institutional mechanism to fulfill the multidimensional needs of older adults through daily living assistance and psychosocial support. By delivering accessible care services and structured social engagement, CACS alleviate familial care giving burdens and mitigate adverse outcomes associated with insufficient family support, such as health deterioration and psychological distress [22,23]. Empirical studies have further demonstrated that CACS implementation correlates with measurable improvements in geriatric health indicators. The nexus between health and subjective well-being is well-established [16,24]. Enhanced health status not only elevates quality of life but also functions as a determinant of holistic well-being in aging populations [25].

Notably, self-rated health, a subjective assessment of one’s health status, has been shown to exert stronger influence on healthcare-seeking behaviors and wellness management than objective clinical metrics [26,27]. Self-rated health also mediates life satisfaction and self-esteem, thereby serving as a predictor of overall well-being of older adults [28,29]. Building on these evidence, we propose the following hypotheses:

H2A: Self-rated health mediates the relationship between CACS and the subjective well-being of older adults.

Within the stress-buffering theoretical architecture, social support may reduce perceived stress by enhancing psychological resilience, CACS, as an institutionalized social support system, may be positively associated with better mental health, potentially through its role in enhancing older adults’ sense of control [30]. And, empirical studies have further demonstrated that mental health disorders significantly impair well-being in older adults, underscoring the necessity of addressing psychological comorbidities in aged care frameworks [31,32]. Building on these evidence, we propose the following hypotheses:

H2B: Mental health mediates the relationship between CACS and the subjective well-being of older adults.

**Fig 1:**
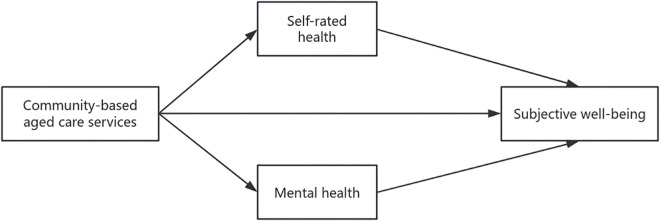
Research hypothesis framework.

The framework diagram of the research hypothesis (Fig 1) is as follows.

## 3 Methods

### 3.1 Data sources

The study utilized data from CLHLS (2018), a nationally representative longitudinal database designed specifically for the aging population, publicly released by the Center for Healthy Aging and Development at Peking University on April 3, 2020. The dataset contains no personally identifiable information, whose methodological validity has been extensively documented in previous studies [33–35]. The 2018 CLHLS provides granular measures critical to this study, including CACS, subjective well-being of older adults, self-rated health and mental health, which are the basis for the following analysis. The data is available in the supplementary file of the article (S1_Data). The analytical sample was derived based on the following eligibility criteria: (1) individuals aged 60 years or older [36]; and (2) exclusion of observations with missing data on core variables, resulting in a final sample of 6933 respondents. A comparative analysis revealed no systematic differences in key demographics between the included and excluded cases, suggesting a limited risk of selection bias.

### 3.2 Measurement of variables

#### 3.2.1 Dependent variable: Subjective well-being.

The three-dimensional model of subjective well-being defines it as a personal overall evaluation of life and includes three dimensions: life satisfaction, positive emotions and negative emotions [37]. Based on this connotation, subjective well-being in this paper is measured in terms of life satisfaction assessment as well as emotional characteristics.

Life satisfaction was assessed using the CLHLS item: “How do you feel about your life now?” Responses were recorded on a 5-point Likert scale: 1 = very bad, 2 = bad, 3 = fair, 4 = good, and 5 = very good, with higher scores indicating greater life satisfaction. Emotional characteristics were measured through seven items from the CLHLS “Personality and Emotional Traits” module, comprising four positively worded statements and three negatively worded statements. Responses were captured on a 5-point frequency scale: 1 = never, 2 = rarely, 3 = sometimes, 4 = often, and 5 = always. Reverse scoring was applied to negative statements to ensure directional consistency.

To construct a comprehensive and psychometrically sound measure, the scores from the life satisfaction item and the seven emotional characteristic items were summed to form a composite SWB score, with total scores ranging from 8 to 40. A Cronbach’s alpha coefficient of 0.722 was obtained for the eight-item scale, indicating acceptable internal consistency [38]. For robustness checks, we also created a binary version of the subjective well-being score (SWB-1), dichotomized at the sample mean (1 = above mean, 0 = otherwise).

#### 3.2.2 Independent Variable: Community-based aged care services.

CACS refer to services provided at the community level to support older adults, which include daily life care, health care services and spiritual comfort for older adults [5]. Service availability was assessed through the CLHLS item: “What social services are available for older adults in your community?” Respondents reported presence or absence of eight service domains: “daily life care, visiting the doctor (delivering medicine), spiritual comfort (chatting to relieve boredom), daily shopping, organizing social and recreational activities, providing legal aid (defending rights), providing health care knowledge, and dealing with family and neighborly disputes.” Each service domain was dichotomously coded (1 = available, 0 = unavailable). A composite service index was calculated by summing affirmative responses, yielding a continuous measure ranging from 0 (no services) to 8 (full-service coverage). This approach captures the diversity and comprehensiveness of community service infrastructure, with higher scores indicating a more robust service environment.

It is important to note that this composite index serves as a proxy for the availability or supply of services within the community, reflecting supply-side infrastructure. It does not capture critical dimensions such as service quality, intensity or frequency of use, or individual utilization by respondents. This measurement focus is a recognized limitation of the dataset and is common in large-scale surveys. The approach, focusing on the breadth of available services, is consistent with prior research and provides a foundational measure of community service infrastructure [39].

#### 3.2.3 Mediating Variables: self-rated health and mental health.

Self-rated health was operationalized as older adults’ subjective evaluation of their holistic health status [40]. This construct was measured through the CLHLS item: “How do you feel about your current health status?” Responses were captured on a 5-point Likert scale:1 = Very bad, 2 = Bad, 3 = Fair, 4 = Good, 5 = Very good.

Mental health was assessed using the 9-item Depression Scale (score range: 9–45) and the 7-item Anxiety Scale (score range: 0–21) from the CLHLS questionnaire with higher scores on both indicating better mental health [41,42]. The decision to integrate these scales into a composite measure was grounded in both theoretical and empirical considerations. Theoretically, it aligns with psychological models that posit a common “psychological distress” dimension underpinning both depression and anxiety [43]. Empirically, we conducted a factor analysis to test this assumption. The results revealed a moderately strong correlation between the depression and anxiety factors (r = 0.594, *p* < 0.001), supporting the existence of a shared latent construct. Furthermore, the combined 16 items demonstrated high internal consistency (Cronbach’s α = 0.856). Based on these evidences, we created a composite mental health variable by summing the standardized scores of both scales (range: 9–66), where higher scores represent better psychological well-being.

#### 3.2.4 Control variables.

The selection of control variables was guided by established determinants of well-being in old age [44]. The control variables in this paper are categorized into three main groups: household demographic characteristics (gender, age, ethnicity, residence, education, marital status, living alone), economic characteristics (relative economic level, adequacy of sources of livelihood, availability of pension insurance), and health characteristics (Sleep quality, ADL).

The definitions of all the study variables are given in Table 1.

**Table 1:** Definitions of study variables.

Variables	Symbols	definitions
**Dependent variables**		
Subjective well-being	SWB	8-40, positively represents the degree of subjective well-being of older adults
	SWB-1	1 = high, 0 = low
**Independent variables**		
Community-based aged care services	CACS	0-8, indicates the number of CACS available
Daily life care	CACS-1	1 = available, 0 = unavailable
home-based medical visits	CACS-2	1 = available, 0 = unavailable
Spiritual comfort	CACS-3	1 = available, 0 = unavailable
Daily shopping	CACS-4	1 = available, 0 = unavailable
social activities	CACS-5	1 = available, 0 = unavailable
legal aid	CACS-6	1 = available, 0 = unavailable
Health knowledge dissemination	CACS-7	1 = available, 0 = unavailable
dispute mediation	CACS-8	1 = available, 0 = unavailable
**Mediating variables**		
Self-rated health	SRH	1-5, positively represents older adults’ self-rated health
Mental health	MH	9-66, positively represents older adults’ mental health
**Control variables**		
Gender	Gender	1 = male, 0 = female
Age	Age	60-110, true age of respondents
Ethnicity	Ethnicity	1 = Han, 0 = other
Place of residence	Residence	1 = town, 0 = village
Education years	Education	0-29, respondents’ years of education
Living alone	Living	1 = living alone, 0 = not living alone
Marital status	Marriage	1 = married and spouse living, 0 = other
Relative economic level	Economics	1 = good, 0 = poor
Adequacy of sources of livelihood	Sources of livelihood	1 = sufficient, 0 = insufficient
Availability of pension insurance	Pension insurance	1 = yes, 0 = no
Sleep quality	Sleeping	1 = good, 0 = poor
Activities of daily living	ADL	1 = good, 0 = poor

### 3.3 Empirical methods

The CLHLS employs a targeted random sampling design. To facilitate comparisons across age groups while controlling for broad environmental confounders, the survey interviews centenarians and, for each, randomly selects approximately 1.5 “proximity-based” younger-old adults (aged 65–79) from the same village/street or county/city, ensuring balanced gender distribution. This design ensures that respondents share similar socio-environmental contexts, making it particularly advantageous for examining age- and gender-related patterns in health and well-being. Regarding the specific design of the CLHLS and our analytical objectives, our analysis was conducted without sampling weights. This decision was made because our primary goal is to examine the theoretical relationships between variables rather than to produce population parameter estimates. Unweighted regression is appropriate for hypothesis testing of this nature, as it avoids the potential inefficiency and bias introduced by weights when the model is correctly specified.

The study utilized STATA 15.1 for empirical analysis, and the empirical analysis code is available in the supplementary information (S2_Code for empirical analysis). First, descriptive statistics were computed for all study variables. Second, bivariate correlation analysis was conducted to assess the relationships between CACS, self-rated health, mental health, and subjective well-being of older individuals. Third, given that our composite subjective well-being score is a continuous variable, a pooled Ordinary Least Squares (OLS) regression model was employed as the primary method to examine its relationship with CACS. This approach is standard and appropriate for modeling continuous outcomes [45]. To facilitate effect‑size comparisons across variables measured on different scales, all regression tables present unstandardized (b*) alongside standardized (*β*) coefficients. *β* indicates the standard‑deviation change in the outcome per standard‑deviation increase in the predictor, providing an intuitive gauge of each predictor’s relative importance. Additionally, to ensure our results were not an artifact of the chosen statistical method, the results were tested for robustness using binary logistic regression models.

Finally, stepwise regression and bias-corrected nonparametric percentile bootstrap method were used to test the mediating effect. Based on the original data (N = 6933), 5000 bootstrap samples were drawn by repeated random sampling. Confidence intervals for indirect effects were constructed by ranking the resulting estimates and statistical significance was determined using 95% deviation correction intervals [46,47]. Indirect effects were considered significant if the confidence intervals did not include zero according to the established thresholds [48]. It is important to note that this mediation analysis is based on theoretical assumptions about the directional relationships between variables. Self-rated health and mental health were specified as mediators based on prior theory and empirical evidence, rather than on observed temporal ordering in the data. Given the cross-sectional design, the mediated effects should be interpreted as statistical decompositions of associations, rather than evidence of temporal or causal processes.

## 4 Results analysis

### 4.1 Descriptive statistics

Analysis of 6933 older adults (mean age: 82.9; 43% male; 50% urban) revealed a moderate level of subjective well-being (mean = 30.83) and a generally low availability of community-based aged care services (mean = 1.73).

Significant disparities were observed in the provision of specific CACS. Health knowledge dissemination (CACS-7) was the most available service (41%), followed by legal aid (CACS-6, 31%) and dispute mediation (CACS-8, 31%). In contrast, coverage of essential daily living supports was severely inadequate: daily life care (CACS-1, 8%), daily shopping assistance (CACS-4, 10%), spiritual comfort (CACS-3, 12%), and social activities (CACS-5, 19%). Even home-based medical visits (CACS-2), a core medical need, reached only 35% of older adults.

Table 2 presents the descriptive statistics for all variables used in this study.

**Table 2:** Descriptive statistics.

Variables	N	Mean	S.D.	Min	Max
**SWB**	6933	30.830	3.73	11	40
**SWB-1**	6933	0.930	0.260	0	1
**CACS**	6933	1.730	2.030	0	8
**CACS-1**	6933	0.080	0.270	0	1
**CACS-2**	6933	0.350	0.480	0	1
**CACS-3**	6933	0.120	0.330	0	1
**CACS-4**	6933	0.100	0.300	0	1
**CACS-5**	6933	0.190	0.390	0	1
**CACS-6**	6933	0.180	0.380	0	1
**CACS-7**	6933	0.410	0.490	0	1
**CACS-8**	6933	0.310	0.460	0	1
**SRH**	6933	3.470	0.890	1	5
**MH**	6933	53.750	7.380	11	66
**Gender**	6933	0.430	0.490	0	1
**Age**	6933	82.900	11.360	60	110
**Ethnicity**	6933	0.920	0.270	0	1
**Residence**	6933	0.500	0.500	0	1
**Education**	6933	2.820	3.590	0	29
**Living**	6933	0.180	0.380	0	1
**Marriage**	6933	0.440	0.500	0	1
**Economics**	6933	0.180	0.380	0	1
**Sources of livelihood**	6933	0.850	0.360	0	1
**Pension insurance**	6933	0.380	0.490	0	1
**Sleeping**	6933	0.530	0.500	0	1
**ADL**	6933	0.700	0.460	0	1

### 4.2 Binary correlations among key variables

Binary correlations between key variables were calculated to quantify the pairwise relationships between them. The results given in Table 3 show that CACS, self-rated health, and mental health were significantly correlated with subjective well-being. CACS were significantly associated with self-rated health and mental health. Finally, there was a significant correlation between self-assessed health and mental health.

**Table 3:** Correlation analysis of key variables.

Variables	SWB	CACS	SRH	MH
**SWB**	1.000			
**CACS**	0.091***	1.000		
**SRH**	0.474***	0.053***	1.000	
**MH**	0.645***	0.060***	0.417***	1.000

Note: *, **and***indicate significance at 10%, 5% and 1%, respectively.

### 4.3 The association between CACS and subjective well-being

The association between CACS and subjective well-being among older adults was estimated using the following ordinary least squares (OLS) model:

To address potential heteroskedasticity, indicated by White’s test (χ²(93) = 125.83, *p* = 0.013), we report robust standard errors in all regressions. Similarly, to assess multicollinearity, we calculated Variance Inflation Factors (VIFs), finding a mean of 1.19 and a maximum of 1.73, confirming the absence of severe multicollinearity.

The regression results are presented in Table 4. Model (1), which included no control variables, established a positive association between CACS and the subjective well-being of older adults. Sequentially adding controls for household demographic characteristics in Model (2), economic status in Model (3), and health status in Model (4) confirmed the robustness of this relationship. The Model (4) indicates that a one-unit increase in the CACS index is associated with a significant 0.117-point increase in subjective well-being (*b* = 0.117, *p* < 0.001).

**Table 4:** The association between CACS and subjective well-being.

Variables	Model (1)	Model (2)	Model (3)	Model (4)
**SWB**	**SWB**	**SWB**	**SWB**
**CACS**	0.159*** (0.017) [0.091]	0.110*** (0.019) [0.062]	0.118*** (0.022) [0.064]	0.117*** (0.022) [0.063]
**Gender**		−0.014 (0.087) [-0.002]	−0.065 (0.094) [-0.009]	−0.275** (0.092) [-0.037]
**Age**		−0.001 (0.004) [-0.004]	−0.007 (0.005) [-0.022]	0.002 (0.005) [0.007]
**Ethnicity**		0.634*** (0.148) [0.042]	0.603*** (0.149) [0.044]	0.540*** (0.145) [0.039]
**Residence**		−0.175* (0.084) [-0.022]	−0.289*** (0.087) [-0.039]	−0.257** (0.084) [-0.034]
**Education**		0.092*** (0.011) [0.107]	0.103*** (0.014) [0.099]	0.096*** (0.014) [0.092]
**Living**		−0.077 (0.120) [-0.007]	−0.001 (0.127) [-0.000]	0.031 (0.122) [0.003]
**Marriage**		−0.148 (0.104) [-0.019]	−0.110 (0.111) [-0.015]	−0.082 (0.108) [-0.011]
**Economics**			1.196*** (0.114) [0.122]	0.958*** (0.112) [0.098]
**Sources of livelihood**			1.870*** (0.127) [0.178]	1.510*** (0.124) [0.144]
**Pension insurance**			0.464*** (0.089) [0.060]	0.481*** (0.087) [0.063]
**Sleeping**				1.554*** (0.086) [0.208]
**ADL**				0.986*** (0.095) [0.121]
**Constant**	30.695*** (0.049)	30.125*** (0.408)	28.616*** (0.450)	26.802*** (0.457)
**N**	10441	8974	6951	6933
**R** ^**2**^	0.008	0.018	0.084	0.143

Note: Coefficients reported as b* (robust SE) [*β*]; *, **and***indicate significance at 10%, 5% and 1%, respectively.

Further analysis examined the effects of individual service components, with results displayed in Table 5. A pronounced heterogeneity in effectiveness was observed. Service CACS-1 demonstrated the strongest effect (*b* = 0.951, *p* < 0.001), followed by CACS-5 (*b* = 0.697, *p* < 0.001). In contrast, the effect of CACS-2 was statistically insignificant (*b* = 0.027, *p* > 0.1), and CACS-8 exhibited only a marginally significant, weak effect (*b* = 0.184, *p* < 0.1). These findings underscore that the benefits of community aged care are highly dependent on the specific type of service provided.

**Table 5:** The association between specific CACS and subjective well-being.

Variables	Model (1)	Model (2)	Model (3)	Model (4)	Model (5)	Model (6)	Model (7)	Model (8)
	SWB	SWB	SWB	SWB	SWB	SWB	SWB	SWB
**CACS-1**	0.951 *** (0.161) [0.070]							
**CACS-2**		0.027 (0.087) [0.004]						
**CACS-3**			0.595 *** (0.131) [0.052]					
**CACS-4**				0.611 *** (0.146) [0.049]				
**CACS-5**					0.697 *** (0.112) [0.073]			
**CACS-6**						0.382 *** (0.110) [0.039]		
**CACS-7**							0.346 *** (0.085) [0.046]	
**CACS-8**								0.184* (0.092) [0.023]
**Control variables**	Yes	Yes	Yes	Yes	Yes	Yes	Yes	Yes
**Constant**	26.904 *** (0.457)	26.937 *** (0.459)	26.915 *** (0.458)	26.849 *** (0.459)	26.848*** (0.457)	26.922*** (0.458)	26.823*** (0.459)	26.900*** (0.458)
**N**	6933	6933	6933	6933	6933	6933	6933	6933
**R** ^**2**^	0.143	0.139	0.141	0.141	0.144	0.140	0.141	0.139

Note: Coefficients reported as b* (robust SE) [*β*]; *, **and***indicate significance at 10%, 5% and 1%, respectively.

Other control variables also have a significant effect on the subjective well-being of older adults, especially the economic characteristics variables and the health characteristics variables. Subjective well-being was higher among older adults who had higher relative economic levels, enough money to live on, and pension insurance, and subjective well-being was higher among older adults who slept better and had no limitations on their ability to perform daily living tasks. Demographic variables such as gender, ethnicity, place of residence, and level of education were also significantly associated with the subjective well-being of older people.

### 4.4 Robustness test

Robustness test was performed in this study by replacing the dependent variable and regression model. Specifically, we operationalized SWB as a binary variable (SWB-1) and tested it using a binary logistic regression model. The regression results are presented in Table 6. The significantly positive CACS coefficients at the 5% level suggest a positive relationship between CACS and the subjective well-being of older adults. These results are consistent with previous results, indicating that the findings are still robust and further support the conclusions of this study. Therefore, hypothesis 1 is valid.

**Table 6:** Regression results of robustness test (Logit Model).

Variables	Model (1)	Model (2)
	SWB-1	SWB-1
**CACS**	0.057** (0.017)	0.084** (0.026)
**Control variables**	No	Yes
**Constant**	2.460*** (0.048)	0.906* (0.523)
**N**	6933	6933

Note: *, **and***indicate significance at 10%, 5% and 1%, respectively.

### 4.5 Heterogeneity analysis

We also examined the heterogeneous effects of gender, age and residence. Columns (1, 2) of Table 7 show the results for gender, columns (3, 4) show the results for age, and columns (5, 6) show the results for residence.

**Table 7:** Heterogeneity analysis results.

Variables	Gender difference		Age difference		Residence difference	
	Male	Female	Younger	Advanced	urban	rural
	(1)	(2)	(3)	(4)	(5)	(6)
**CACS**	0.059* (0.032) [0.033]	0.156*** (0.029) [0.083]	0.132*** (0.032) [0.073]	0.104*** (0.029) [0.056]	0.068* (0.030) [0.036]	0.165*** (0.031) [0.091]
**Control variables**	Yes	Yes	Yes	Yes	Yes	Yes
**Constant**	27.043*** (0.679)	26.362*** (0.631)	26.913*** (0.305)	26.904*** (0.256)	26.926*** (0.285)	26.859*** (0.251)
**N**	2969	3964	3094	3839	3495	3438
**R** ^**2**^	0.131	0.153	0.135	0.157	0.138	0.150
**Chow test** **P-Value**	0.0039***		0.0263**		0.615	

Note: Coefficients reported as b* (robust SE) [*β*]; *, **and***indicate significance at 10%, 5% and 1%, respectively.

Heterogeneity analysis reveals significant disparities in the association between CACS and subjective well-being across subpopulations. A notable gender disparity exists, with the effect significantly stronger for older women (*b* = 0.156, *p* < 0.001) than for men (*b* = 0.059, *p* < 0.1), a difference confirmed by a Chow test [49]. Similarly, a significant age-stratified gradient was observed, where the association was more pronounced among younger older adults (aged 60–80) compared to their advanced-age counterparts (80+), with the Chow test validating this differential effect (*p* = 0.0263). In terms of residence, although the Chow test was not statistically significant, the point estimate for rural elders (*b* = 0.165, *p* < 0.001) was substantially larger than that for urban residents (*b* = 0.068, *p* < 0.1), the considerable magnitude of the coefficient gap suggests that CACS may have a uniquely potent association with well-being in resource-scarce rural areas, where formal support alternatives are often limited.

### 4.6 Test and comparison of the mediating effects

According to the previous study [50], we tested and compared the mediating effects in two steps, the first step used stepwise regression method to test the effect of CACS on older people’s self-rated health and mental health, and the effect of self-rated health and mental health on their subjective well-being, and Table 8 gives the results. And then the bootstrap mediating effect decomposition method was used to test the robustness of the mediating effects. We resampled the sample 5000 times with put-backs to construct confidence intervals for the mediating effects, and also calculated the proportion of the intermediary effects of self-rated health and mental health in the total effects, and the results are shown in Table 9.

**Table 8:** Association between CACS and Self-Rated and Mental Health.

Variables	Model (1)	Model (2)	Model (3)	Model (4)
	SRH	MH	SWB	SWB
**CACS**	0.022*** (0.005) [0.049]	0.152*** (0.040) [0.042]	0.081*** (0.020) [0.044]	0.072*** (0.017) [0.039]
**SRH**			1.674*** (0.052) [0.398]	
**MH**				0.300*** (0.006) [0.594]
**Control variables**	Yes	Yes	Yes	Yes
**Constant**	2.103*** (0.107)	43.869*** (0.931)	23.282*** (0.434)	13.628*** (0.465)
**N**	6933	6933	6933	6933
**R** ^**2**^	0.177	0.192	0.273	0.428

Note: Coefficients reported as: b* (robust SE) [*β*]; *, **and***indicate significance at 10%, 5% and 1%, respectively.

**Table 9:** Test and comparison of the mediating effects.

Variables	[95% CI]	Direct effect	Indirect effect	Total effect	Proportion of direct effect	Proportion of indirect effect
**Model (1)**	[0.020][0.052]	0.081	0.036	0.117	69.23%	30.77%
**SRH**						
**Model (2)**	[0.022][0.069]	0.072	0.045	0.117	61.54%	38.46%
**MH**						

As demonstrated in Table 8, Models 1 and 2 indicate that a higher CACS score shows significant positive associations with both better self-rated health and mental health among older adults. This suggests that CACS implementation is positively associated with self-rated health and mental health among community-dwelling older populations. Subsequent analyses in Models (3) and (4) revealed: a persistent direct association between CACS and subjective well-being older adults. Meanwhile, self-rated health and mental health have a significant positive effect on subjective well-being. In other words, the better the self-rated health and mental health among older adults, the higher the subjective well-being. The mediating roles of self-rated health and mental health in the association between CACS and subjective well-being among older adults were preliminarily supported.

In Model (1) of Table 9, Bootstrap 95% confidence interval is ([0.020], [0.052]) without 0. In Model (2), Bootstrap 95% confidence interval is ([0.022], [0.069]) without 0, which suggests that the mediating effect is significant at 95% confidence level. The findings confirm the existence of dual mediation pathways through which CACS is associated with subjective well-being among older adults. with both self-rated health and mental health serving as empirically substantiated psychological mechanisms. Therefore, Hypothesis 2A and Hypothesis 2B are valid.

Furthermore, in Model (1), the total effect decomposition demonstrated that 69.23% of CACS’ influence on older adults’ subjective well-being operated through direct pathways, while self-rated health mediated 30.77% of the observed effects. Model (2) exhibited comparable effect distribution patterns, with direct effects accounting for 61.54% and mental health mediating 38.46% of the total impact. Comparing the proportions of the mediating effects of self-rated health and mental health in the total effect, it can be concluded that the mediating effect of mental health is slightly higher than that of self-rated health in the pathway of CACS on subjective well-being in older adults.

## 5 Discussion

The well-being of older adults is intricately linked to societal stability and sustainable development. To address the challenges posed by rapid population aging, the development of community-based aged care services has become a significant public health priority. Utilizing nationally representative data from the CLHLS (2018), this study investigated the association between the availability of community-based aged care services (CACS) and the subjective well-being of older adults in China. Our analysis further explored potential psychosocial pathways through self-rated health and mental health, while controlling for key sociodemographic covariates.

A primary finding of this study is the observed positive association between the availability of CACS and higher levels of subjective well-being among older adults. Crucially, our disaggregated analysis revealed substantial heterogeneity in the effects of specific services. Our findings align with and extend the healthy aging paradigm, suggesting three potential pathways: optimized quality-of-life metrics, amplified life satisfaction coefficients, and facilitated successful aging trajectories [10–12]. To understand these associations, social support theory offers a valuable framework, positing that multidimensional social resources (including emotional sustenance and instrumental help) can be associated with reduced psychological and physiological stress responses [30,51]. Consistent with this view, recent research underscores that community-based services function as a vital source of social support, complementing or supplementing familial networks [14,52]. A rich social support network is linked to individuals with positive emotional experiences and satisfies their psychological needs such as sense of belonging and self-esteem. In the current social context, many older adults are facing the double pressure of “insufficient care under intergenerational separation” and “weakening of the family’s function in old-age care”, and it is difficult to satisfy the old-age needs of older adults in the traditional family old-age care model, in which the children provide care for older adults. CACS, as a form of socialized aged care, is associated with higher subjective well-being among older adults, and this association may be partly explained by the life care, emotional comfort, and social interaction opportunities it provides.

The mediation analysis identified self-rated health and mental health as significant mediators, reflecting complementary pathways. However, reverse causality cannot be ruled out; subjective well-being may also influence self-rated health and mental health perceptions. Thus, these pathways should be interpreted as associations consistent with theory, not causal evidence. From a cognitive perspective, social cognitive theory suggests that self-perception of personal circumstances serves as a cognitive mediator, systematically shaping affective states and behavioral patterns through self-regulatory mechanisms [53]. When older people receive CACS, the services are associated with their health status, including the maintenance of bodily functions. Subsequent improvements in, or maintenance of, health can foster a positive self-assessment of health, and this positive self-assessment is correlated with higher subjective well-being [26,27,29].

From an affective perspective, psychological stress theory suggests that older adults are susceptible to stress from life changes—such as physical decline, social isolation, or loss of loved ones—which can trigger negative emotions like depression and anxiety, thereby diminishing subjective well-being [54]. CACS, particularly those offering structured social activities and psychological support, may help alleviate these negative emotions by providing engagement opportunities and emotional comfort [55,56]. Such positive engagements are linked to positive emotional experiences, which may buffer against negative emotions and contribute to better mental health, ultimately associated with higher subjective well-being [57].

These findings offer nuanced insights for policy and practice. First, they suggest that resource allocation should prioritize services with relatively stronger observed associations, such as daily life care and social activities, while recognizing that these effect sizes are modest. Second, the substantial variation in service effects highlights that service quality and targeting are likely more important than simply expanding service variety. Policymakers should conduct careful needs assessments to ensure services match the specific requirements of local elderly populations. Third, the suggested mediating roles of self-rated health and mental health reinforce the need for integrated care models that address both physical and psychological well-being simultaneously.

Several limitations of this study must be acknowledged. First and foremost, the cross-sectional nature of the data precludes definitive causal inference. While we observe significant associations, it is important to emphasize that these relationships could be bidirectional. Furthermore, despite our efforts to control for a comprehensive set of covariates, the possibility of unobserved confounding variables influencing the observed associations cannot be ruled out. Second, our measure of CACS was limited to the availability of services, without capturing critical dimensions such as service quality, frequency of use, or user satisfaction. Consequently, our findings should not be interpreted as evidence that using CACS directly improves well-being, but rather that the presence of such services in a community is positively associated with well-being. Third, the generalizability of our findings may be constrained by the specific characteristics of our sample, particularly the advanced mean age. Future research should employ longitudinal designs and incorporate measures of service quality, frequency of use, and user satisfaction to better understand how CACS relate to well-being. Examining whether these associations vary across different subpopulations of older adults would also inform more targeted policy interventions.

## 6 Conclusion

In conclusion, this study provides evidence that community-based aged care services (CACS) are positively associated with the subjective well-being of older adults in China. The analysis reveals three key findings. First, CACS demonstrates a significant positive association with seniors’ subjective well-being, suggesting that greater access to these services may correspond with more favorable evaluations of life among older adults. Second, this relationship operates through dual mediation pathways of self-rated health and mental health. Third, a comparative analysis indicates that the mediating effect of mental health is stronger than that of self-rated health. Furthermore, the strength of this association varies across specific service types, with relatively stronger links observed for services addressing daily living needs and social activities. These findings suggest that initiatives should not only aim to expand the coverage of CACS but also prioritize the quality and accessibility of services. Prioritizing resource allocation towards services with relatively stronger associations, such as daily living assistance and health literacy programs, may help maximize well-being outcomes.

## Supporting information

S1 File Data. (XLSX)

S2 File Code for empirical analysis. (DOCX)

S3 File Detailed variables documentation. (DOCX)
