# Supplementary material for: Associations between community-based aged care services and subjective well-being among older adults in China: the mediating roles of self-rated and mental health
Source: PLoS One. 2026 Apr 6;21(4):e0341877. doi: 10.1371/journal.pone.0341877 (PMC13052906; doi:10.1371/journal.pone.0341877)
Supplement: S2 File — (DOCX) [file pone.0341877.s002.docx]

***Descriptive statistics**

gen complete_sample = !missing(SWB, CACS, Gender, Age, Ethnicity, Residence, Education, Living, Marriage, Economics, Sources_of_livelihood, Pension_insurance, Sleeping, ADL, f141, f142, f143, f144, f145, f146, f147, f148)

estpost summarize SWB CACS Gender Age Ethnicity Residence Education Living Marriage Economics Sources_of_livelihood Pension_insurance Sleeping ADL f141 f142 f143 f144 f145 f146 f147 f148 if complete_sample==1

***Correlation analysis of key variables.**

asdoc pwcorr_a SWB CACS Self_rated_health Mental_health

***Multicollinearity test**

regress SWB CACS Gender Age Ethnicity Residence Education Living Marriage Economics Sources_of_livelihood Pension_insurance Sleeping ADL

estat vif

***Heteroscedasticity test**

estathettest

estat imtest, white

***The association between CACS and subjective well-being**

regress SWB CACS, vce(robust)//model(1)

regress SWB CACS Gender Age Ethnicity Residence Education Living Marriage, vce(robust)// model(2)

regress SWB CACS Gender Age Ethnicity Residence Education Living Marriage Economics Sources_of_livelihood Pension_insurance, vce(robust)// model(3)

regress SWB CACS Gender Age Ethnicity Residence Education Living Marriage Economics Sources_of_livelihood Pension_insurance Sleeping ADL, vce(robust)// model(4)

***The association between specific CACS and subjective well-being**

local services "f141 f142 f143 f144 f145 f146 f147 f148"

foreach service of local services {

regress SWB `service' Gender Age Ethnicity Residence Education Living Marriage Economics Sources_of_livelihood Pension_insurance Sleeping ADL, vce(robust)

***Result Output**

outreg2 using result.doc,replace tstat bdec(3)tdec(2)

***Robustness test**

logit SWB_1 CACS, vce(robust)

logit SWB_1 CACS Gender Age Ethnicity Residence Education Living Marriage Economics Sources_of_livelihood Pension_insurance Sleeping ADL i.yearin, vce(robust)

***Heterogeneity analysis**

**//Gender**

reg SWB CACS Age Ethnicity Residence Education Living Marriage Economics Sources_of_livelihood Pension_insurance Sleeping ADL if Gender==1

reg SWB CACS Age Ethnicity Residence Education Living Marriage Economics Sources_of_livelihood Pension_insurance Sleeping ADL if Gender==0

**//Age**

reg SWB CACS Gender Ethnicity Residence Education Living Marriage Economics Sources_of_livelihood Pension_insurance Sleeping ADL if age==1

reg SWB CACS Gender Ethnicity Residence Education Living Marriage Economics Sources_of_livelihood Pension_insurance Sleeping ADL if age==0

**//Residence**

reg SWB CACS Gender Ethnicity Residence Education Living Marriage Economics Sources_of_livelihood Pension_insurance Sleeping ADL if Residence==1

reg SWB CACS Gender Ethnicity Residence Education Living Marriage Economics Sources_of_livelihood Pension_insurance Sleeping ADL if Residence==0

**//CHOW TEST**

chowtest SWB CACS Age Ethnicity Residence Education Living Marriage Economics Sources_of_livelihood Pension_insurance Sleeping ADL,group(Gender)

chowtest SWB CACS Gender Ethnicity Residence Education Living Marriage Economics Sources_of_livelihood Pension_insurance Sleeping ADL,group(age)

chowtest SWB CACS Gender Ethnicity Residence Education Living Marriage Economics Sources_of_livelihood Pension_insurance Sleeping ADL,group(Residence)

***Test and comparison of the mediating effects**

**//stepwise regression**

reg Self_rated_health CACS Gender Age Ethnicity Residence Education Living Marriage Economics Sources_of_livelihood Pension_insurance Sleeping ADL,vce(robust)

reg Mental_health CACS Gender Age Ethnicity Residence Education Living Marriage Economics Sources_of_livelihood Pension_insurance Sleeping ADL,vce(robust)

reg SWB CACS Self_rated_health Gender Age Ethnicity Residence Education Living Marriage Economics Sources_of_livelihood Pension_insurance Sleeping ADL,vce(robust)

reg SWB CACS Mental_health Gender Age Ethnicity Residence Education Living Marriage Economics Sources_of_livelihood Pension_insurance Sleeping ADL,vce(robust)

**//Bootstrap**

Bootstrap r(ind_eff)r(dir_eff),reps(5000):sgmediation SWB,mv(Self_rated_health)iv(CACS)cv(Gender Age Ethnicity Residence Education Living Marriage Economics Sources_of_livelihood Pension_insurance Sleeping ADL)

bootstrap r(ind_eff)r(dir_eff),reps(5000):sgmediation SWB,mv(Mental_health)iv(CACS)cv(Gender Age Ethnicity Residence Education Living Marriage Economics Sources_of_livelihood Pension_insurance Sleeping ADL)
