# Supplementary material for: Associations between community-based aged care services and subjective well-being among older adults in China: the mediating roles of self-rated and mental health
Source: PLoS One. 2026 Apr 6;21(4):e0341877. doi: 10.1371/journal.pone.0341877 (PMC13052906; doi:10.1371/journal.pone.0341877)
Supplement: S3 File — (DOCX) [file pone.0341877.s003.docx]

**Measurement of Variables**

**Dependent variable: Subjective Well-Being**

The three-dimensional model of subjective well-being defines it as a personal overall evaluation of life and includes three dimensions: life satisfaction, positive emotions and negative emotions [1]. Based on this connotation, subjective well-being in this paper is measured in terms of life satisfaction assessment as well as emotional characteristics.

Life satisfaction was assessed using the CLHLS item: “How do you feel about your life now?” Responses were recorded on a 5-point Likert scale: 1 = very bad, 2 = bad, 3 = fair, 4 = good, and 5 = very good, with higher scores indicating greater life satisfaction. Emotional characteristics were measured through seven items from the CLHLS “Personality and Emotional Traits” module, comprising four positively worded statements and three negatively worded statements. Responses were captured on a 5-point frequency scale: 1 = never, 2 = rarely, 3 = sometimes, 4 = often, and 5 = always. Reverse scoring was applied to negative statements to ensure directional consistency.

To construct a comprehensive and psychometrically sound measure, the scores from the life satisfaction item and the seven emotional characteristic items were summed to form a composite SWB score, with total scores ranging from 8 to 40. A Cronbach’s alpha coefficient of 0.722 was obtained for the eight-item scale, indicating acceptable internal consistency [2]. For robustness checks, we also created a binary version of the subjective well-being score (SWB-1), dichotomized at the sample mean (1 = above mean, 0 = otherwise).

**Independent Variable: Community-based aged care services**

CACS refers to the community as the basic unit to provide various forms of services and assistance to older adults, which include daily life care, health care services and spiritual comfort for older adults [3]. Service availability was assessed through the CLHLS item: “What social services are available for older adults in your community?” Respondents reported presence or absence of eight service domains: “daily life care, visiting the doctor (delivering medicine), spiritual comfort (chatting to relieve boredom), daily shopping, organizing social and recreational activities, providing legal aid (defending rights), providing health care knowledge, and dealing with family and neighborly disputes.” Each service domain was dichotomously coded (1 = available, 0 = unavailable). A composite service index was calculated by summing affirmative responses, yielding a continuous measure ranging from 0 (no services) to 8 (full-service coverage). This approach captures the diversity and comprehensiveness of community service infrastructure, with higher scores indicating a more robust service environment.

It is important to note that this composite index serves as a proxy for the availability or supply of services within the community. It does not capture critical dimensions such as the quality of services, their accessibility to individual older adults or the respondents' actual utilization of these services. This measurement focus is a recognized limitation of the dataset and is common in large-scale surveys. The approach, focusing on the breadth of available services, is consistent with prior research and provides a foundational measure of community service infrastructure [4].

**Mediating Variables: self-rated health and mental health**

Self-rated health was operationalized as older adults’ subjective evaluation of their holistic health status [5]. This construct was measured through the CLHLS item: “How do you feel about your current health status?” Responses were captured on a 5-point Likert scale:1 = Very bad, 2 = Bad, 3 = Fair, 4 = Good, 5 = Very good.

Mental health was assessed using the 9-item Depression Scale (score range: 9-45) and the 7-item Anxiety Scale (score range: 0-21) from the CLHLS questionnaire with higher scores on both indicating better mental health [6, 7]. The decision to integrate these scales into a composite measure was grounded in both theoretical and empirical considerations. Theoretically, it aligns with psychological models that posit a common "psychological distress" dimension underpinning both depression and anxiety [8]. Empirically, we conducted a factor analysis to test this assumption. The results revealed a moderately strong correlation between the depression and anxiety factors (r = 0.594, p < 0.001), supporting the existence of a shared latent construct. Furthermore, the combined 16 items demonstrated high internal consistency (Cronbach’s α = 0.856). Based on this evidence, we created a composite mental health variable by summing the standardized scores of both scales (range: 9-66), where higher scores represent better psychological well-being.

**Control Variables**

The selection of control variables was guided by established determinants of well-being in old age [9]. The control variables in this paper are categorized into three main groups: household demographic characteristics (gender, age, ethnicity, residence, education, marital status, living alone), economic characteristics (relative economic level, adequacy of sources of livelihood, availability of pension insurance), and health characteristics (Sleep quality, ADL).

**References**

1. Diener E. Subjective well-being. Psychological Bulletin. 1984; 95:542. doi: 10.1037/0033-2909.95.3.542

2. Cronbach L J J P. Coefficient alpha and the internal structure of tests. Psychometrika. 1951; 16:297-334. doi: 10.1007/BF02310555

3. Joling K J, Van Eenoo L, Vetrano D L, Smaardijk V R, Declercq A, Onder G, et al. Quality indicators for community care for older people: A systematic review. Plos One. 2018; 13:doi: 10.1371/journal.pone.0190298

4. Buffel T and Phillipson C. A Manifesto for the Age-Friendly Movement: Developing a New Urban Agenda. Journal of Aging & Social Policy. 2018; 30:173-192. doi: 10.1080/08959420.2018.1430414

5. Desalvo K B, Bloser N, Reynolds K, He J, and Muntner P J J O G I M. Mortality prediction with a single general self-rated health question: a meta-analysis. Journal of general internal medicine. 2006; 21:267-275. doi: 10.1111/j.1525-1497.2005.00291.x

6. Gaitz C M and Scott J. Age and the measurement of mental health. Journal of health and social behavior. 1972; 13:55-67. doi: 10.2307/2136973

7. O'connor M and Casey L. The Mental Health Literacy Scale (MHLS): A new scale-based measure of mental health literacy. Psychiatry Research. 2015; 229:511-516. doi: 10.1016/j.psychres.2015.05.064

8. Watson D. Rethinking the mood and anxiety disorders: a quantitative hierarchical model for DSM-V. Journal of abnormal psychology. 2005; 114:522-36. doi: 10.1037/0021-843x.114.4.522

9. Smith J L, Bihary J G, O'connor D, Basic A, and O'brien C I. Impact of Savoring Ability on the Relationship Between Older Adults' Activity Engagement and Well-Being. Journal of Applied Gerontology. 2020; 39:323-331. doi: 10.1177/0733464819871876
